# Potential pharmacological quality control markers in the traditional Japanese medicine Hangeshashinto: identifying anti-inflammatory ingredients through a cell-based bioassay and multicomponent analysis

**DOI:** 10.1080/13880209.2025.2583068

**Published:** 2025-11-15

**Authors:** Ryota Imai, Yuta Muraki, Akinori Nishi, Katsuya Ohbuchi

**Affiliations:** Research & Development Division, TSUMURA & CO., TSUMURA Advanced Technology Research Laboratories, Ibaraki, Japan

**Keywords:** Kampo, multicomponent drug, Hangeshashinto, inflammation, stomatitis, phenotypic assay, prostaglandin E2, LC-MS, correlation analysis, quality maker

## Abstract

**Context:**

The traditional Japanese medicine Hangeshashinto (HST) is a multicompound drug used for stomatitis. The identification of HST’s active ingredients is essential for understanding its mechanism of action and establishing pharmacological quality control markers, but remains unclear due to its multicomponent drug.

**Objective:**

To systematically explore anti-inflammatory ingredients in HST using a combined approach involving a cell-based bioassay and multicomponent analysis, and to identify potential quality marker compounds.

**Materials and methods:**

A cell-based bioassay modeling stomatitis was established using human oral keratinocytes (HOK), with interleukin (IL)-1β–induced prostaglandin E2 (PGE2) as an inflammatory indicator. The PGE2 inhibitory effects of quality-controlled 101 HST manufacturing lots were compared. Multicomponent analysis was performed on the 101 HST samples, correlating the intensity of 121 component peaks with the pharmacological activities. Ingredients with the highest correlation coefficients were validated for their PGE2 inhibitory effects.

**Results:**

A total of 101 HST samples (30 µg/mL: approximately IC_50_) showed consistent inhibition of IL-1β–induced PGE2 production in HOK (41.33%–67.38% with 100% as IL-1β). Correlation analysis between PGE2 inhibitory activities and peak intensity of 121 components revealed the contribution of each ingredient to the pharmacological effect. Eight ingredients (glycycoumarin, neoglycyrol, [6]-shogaol, [8]-shogaol, [10]-shogaol, [6]-gingerol, [8]-gingerol, and [10]-gingerol) with the highest correlation coefficients (below −0.30) exhibited PGE2-inhibitory potential.

**Conclusion:**

The standardization of current quality control markers contributes to the stability of HST’s anti-inflammatory effects. The eight components discovered by this integrated analysis method may become novel quality control marker ingredients for further improving the pharmacological quality of HST.

## Introduction

Traditional Japanese Kampo medicine, comprising multiple herbal formulations, is approved by the Ministry of Health, Labor, and Welfare, covered by national health insurance programs in Japan, and widely prescribed as a major therapeutic modality in modern clinical practice (Motoo et al. 2011; Kono et al. 2015; Arai and Kawahara 2019). In recent years, the various pharmacological effects of multicomponent Kampo medicines have been elucidated, and identification of the primary active ingredients responsible for their therapeutic effects has been progressing. For example, regarding Kampo medicines such as Daikenchuto, Rikkunshito, and Yokukansan, their primary active ingredients and targets have been identified, and their multi-effect mechanisms are becoming increasingly explainable from a pharmacological perspective (Ikarashi and Mizoguchi 2016; Mizoguchi and Ikarashi 2017a, 2017b; Inokuchi et al. 2021; Yamada et al. 2021; Namiki et al. 2022; Yamaguchi et al. 2023; Kainuma et al. 2024). Furthermore, beyond the mere identification of active ingredients, research on the characteristics of the multicomponent pharmaceuticals—such as studies employing multicomponent analysis and omics technologies—has advanced significantly (Nishi et al. 2017; Yamamoto 2022). In any case, challenges remain in scientifically explaining and verifying the effects of multi-component drugs, and it is essential to elucidate how the active ingredients exert their pharmacological effects within the body.

Kampo medicines, which play an essential role in healthcare, must be provided as high-quality pharmaceuticals. Their quality is tightly regulated, and the medicine is produced in accordance with Japanese good manufacturing practice. The quality of constituent herbs is influenced by environmental factors, including climate, temperature, and soil conditions. Strict control of the many ingredients in crude drug material is recognized as challenging (Yamamoto 2022). To address this, the Japanese Pharmacopeia specifies quality marker ingredients detectable through physicochemical tests to standardize each Kampo medicine (Ministry of Health, Labour and Welfare 2021). These quality markers are typically selected for being characteristic, abundant, stable, and easy to quantify in crude drugs (Arai and Kawahara 2019), and their relationship to pharmacological action remains unclear in many cases. Identifying the primary active ingredients involved in specific pharmacological activities is crucial not only for discovering new mechanisms of action in multicomponent Kampo medicines but also for utilizing the identified active ingredients as candidate quality markers to enable future pharmacologically evidence-based quality design of Kampo medicines. Kampo medicines are expected to act *via* multiple targets owing to their complex composition. Thus, phenotypic assays that assess cellular responses are considered more suitable than single-target assays for evaluating pharmacological effects.

The traditional Japanese medicine Hangeshashinto (HST), composed of seven crude drugs (*Pinellia* tuber, *Scutellaria* root, processed ginger, *Glycyrrhiza*, jujube, ginseng, and *Coptis* rhizome), is prescribed for acute and chronic gastroenteritis, diarrhea or soft stools, dyspepsia, gastroptosis, neurological gastritis, stomach weakness, hangover, belching, heartburn, stomatitis, and neurosis. Clinical studies have shown that HST improves stomatitis caused by anticancer drugs (Matsuda et al. 2015). A foundational study reported that HST suppresses interleukin (IL)-1β–induced prostaglandin E2 (PGE2) production in human oral keratinocytes (HOKs) (Kono et al. 2014). Regarding its mechanism, its ingredients baicalin and berberine inhibit cyclooxygenase (COX)-2 gene expression while wogonin inhibits both *COX-2* expression and p38/JNK phosphorylation, whereas [6]-gingerol and [6]-gingerol inhibit enzymes involved in PGE2 production (Kono et al. 2014). Among these, baicalin and berberine are defined as quality control markers for HST in the Japanese Pharmacopeia (Ministry of Health, Labour and Welfare 2021). However, other anti-inflammatory ingredients have also been reported. Standardizing not only baicalin and berberine but also additional active ingredients is important for ensuring HST’s anti-inflammatory efficacy against stomatitis. Comprehensive evaluation of these ingredients requires establishing a phenotype-focused cell-based assay that can aggregate the responses of multiple compounds, and to objectively select active ingredients to be researched.

In this study, to solve the problem of searching for active ingredients in HST, which has been selected by hypothesis-driven methods, we developed a cell-based bioassay using HOK cells to model oral inflammation, objectively explored for active ingredients using a data-driven approach. In cancer therapy-induced stomatitis, the mechanism has been proposed whereby inflammatory cytokines released from immune cell activation (IL-1β is a representative inflammatory cytokine) damage the oral epithelium (Lalla et al. 2010). Biopsies taken from patients undergoing chemotherapy indicate that COX-2 activity is increased in oral mucosal (Logan et al. 2007), suggesting an increase in PGE2 within the oral cavity of the patients. Furthermore, many studies have demonstrated that HST inhibits COX-2 and PGE2 (Kase et al. 1997; 1998; 1999; Kitamura et al. 2014; Kono et al. 2014; Matsuda et al. 2015; Kato et al. 2016; Kamide et al. 2017; Miyashita et al. 2018), PGE2 was selected as an inflammatory indicator, anticipating the establishment of a cell-based bioassay with low variability. Based on these reports, we selected IL-1β as the stimulant and PGE2 as the inflammatory indicator, establishing a valid bioassay that can be extrapolated to clinical settings. Using this cell-focused assay employing PGE2 as an inflammatory indicator, the anti-inflammatory activities of 101 HST manufacturing lots, standardized by quality marker ingredients (i.e., baicalin, berberine, and glycyrrhizic acid), were compared. Furthermore, we performed a multicomponent analysis of 121 peaks using targeted liquid chromatography–mass spectrometry (LC-MS) analysis and correlated these results with anti-inflammatory activity to identify ingredients potentially responsible for anti-inflammatory effects.

## Materials and methods

### Reagents

HST extract powder (Table S1) was obtained from TSUMURA & CO. (Tokyo, Japan) and manufactured by spray-drying a hot water extract of a mixture comprising seven crude drugs: *Pinellia* tuber (5.0 g), *Scutellaria* root (2.5 g), processed ginger (2.5 g), *Glycyrrhiza* (2.5 g), jujube (2.5 g), ginseng (2.5 g), and *Coptis* rhizome (2.5 g). TSUMURA & CO. also supplied all other Kampo extract powders (Table S2), which were manufactured by spray-drying hot water extracts of crude drug mixtures based on amounts specified in the Japanese Pharmacopeia. The representative HST (rep., Lot# 392107100) was selected by evaluating the quantities of quality marker ingredients (i.e., baicalin, berberine, and glycyrrhizic acid). We chose this lot because the contents of these ingredients were close to the overall mean and within the range of variation (mean ± 1.65 SD) observed among the manufacturing lots produced between 2018 and 2020. Plant materials were authenticated *via* morphology and marker compound analyses following the Japanese Pharmacopeia methods and company standards. The quality of all Kampo extracts was standardized based on Good Manufacturing Practices defined by the Japanese Ministry of Health, Labor, and Welfare.

Diclofenac sodium (DCF; Cat No.: 043–22851) was purchased from Fujifilm Wako Pure Chemical (Osaka, Japan). Baicalin, baicalein, [6]-shogaol, [6]-gingerol, and magnoflorine were obtained from TSUMURA & CO. Schaftoside (Cat No.: HY-N0703), [8]-shogaol (Cat No.: HY-N2435), [10]-shogaol (Cat No.: HY-N2434), and [10]-gingerol (Cat No.: HY-N0448) were purchased from MedChemExpress (NJ, USA). Chrysin 6-C-glucoside 8-C-arabinoside (Cat No.: CFN92285) and neoglycyrol (Cat No.: CFN90792) were obtained from Wuhan ChemFaces Biochemical (Hubei, China). Cayman Chemical (MI, USA) supplied [8]-gingerol (Cat No.: 11841), Fisher Scientific International (NH, USA) provided 2-acetoxy-3-methoxybenzoic acid (Cat No.: 10712693), and Sigma-Aldrich (STL, USA) supplied glycycoumarin (Cat No.: SML3107). All other chemicals were purchased from commercial sources.

### Cell culture

Primary HOKs (species: human, Lot No.: 17787 (Mycoplasma was not detected in this lot), Cat No.: 2610) were purchased from ScienCell Research Laboratories (CA, USA). HOK cells were cultured in Oral Keratinocyte Medium (Cat No.: 2611; ScienCell Research Laboratories) supplemented with 1% Oral Keratinocyte Growth Supplement (Cat No.: 2652; ScienCell Research Laboratories), 1% Penicillin/Streptomycin Solution (Cat No.: 0503; ScienCell Research Laboratories), and 10% heat-inactivated Fetal Bovine Serum (FBS; Lot No. 2353776RP, Cat No.: 16140-071; Thermo Fisher Scientific, MA, USA) using Poly-L-Lysine-coated dishes (Cat No.: 4020-040; AGC Techno Glass, Shizuka, Japan). HOK cells were maintained at 37 °C in a 5% CO_2_ incubator during all experiments.

### PGE2 measurements

All assays were performed using fifth-passage HOK cells. Semi-confluent cells were detached using 0.25% Trypsin/EDTA Solution (Cat No.: 0103; ScienCell Research Laboratories), neutralized using Trypsin Neutralization Solution (Cat No.: 0113; ScienCell Research Laboratories), and collected. Cells were seeded into 96-well Poly-L-Lysine–coated plates (Cat No.: MS-0096L; Sumitomo Bakelite, Tokyo, Japan) at 4–8 × 10^4^ cells/well in Oral Keratinocyte Medium containing 2% FBS (100 μL/well).

After overnight incubation, cells were washed with phosphate-buffered saline and treated with test reagents (100 μL/well) immediately after adding human IL-1β (Cat No.: AF-200-1B; PeproTech, NJ, USA) diluted in serum-free complete medium with or without 0.2% dimethyl sulfoxide (DMSO; 100 μL/well). Vehicle-only treatments were used for blank wells, and control wells contained cells without test compounds. The final volume in each well was 200 μL. Test reagents and IL-1β solutions reached final concentrations upon mixing in each well. Supernatants were collected 24 h after treatment. DCF was diluted in serum-free complete medium with or without 0.2% DMSO. HST and other Kampo extract powders were suspended in serum-free medium and centrifuged, after which the supernatants were diluted for use as test reagents. All single ingredients were dissolved in DMSO and further diluted with serum-free medium. DMSO concentration in assays was adjusted to 0.2%. Vehicles were selected appropriately for each experiment.

PGE2 concentrations in supernatants were measured using an enzyme immunoassay (EIA) kit (Cat No.: 514010; Cayman Chemical) according to the manufacturer’s instructions. Supernatants were first diluted 10-fold with EIA kit buffer, and absorbance was read at 405–420 nm using a Multiskan GO microplate reader (Thermo Fisher Scientific).

To correct for interpolation and interday variability, an internal standard was included in each assay. The PGE2 production rate for each treatment was calculated using the following formula: [(measured value/mean IL-1β value on the same plate) × 100/mean inhibition rate of representative sample (or positive control) on the same plate] × mean inhibition rate of all representative samples (or positive controls).

### Cell viability assay

After supernatants were collected for PGE2 analysis, HOK cell viability was assessed using a Cell Counting Kit-8 (CCK-8; Cat No.: CK04; Dojindo Laboratories, Kumamoto, Japan). A 10% CCK-8 solution in Oral Keratinocyte Medium was added to each well (100 μL/well) and incubated for 2 h at 37 °C. Absorbance at 450 nm was measured using the Multiskan GO microplate reader. Absorbance for each treatment was normalized using IL-1β–treated cells as 100%.

### Multicomponent analysis

*HST extraction method*

HST powder (∼0.2 g; actual range: 0.1975–0.2024 g) was weighed into a screw-top centrifuge tube, and 20 mL of purified water was added. The mixture was shaken at 250 rpm for 30 min, followed by centrifugation at 3,000 rpm for 5 min. The supernatant was then transferred to a glass vial and centrifuged again at 7,000 rpm for 5 min to prepare the sample solution.

*Component selection*

To analyze HST components, analyte peaks were selected from the crude drugs comprising HST (one sample per drug). Each extract was analyzed using a liquid chromatography–charged aerosol detector (LC-CAD), and peaks with content ≥ 0.3 mg/g were selected. The peak selection criterion was set with reference to guideline for International Council for Harmonization of Technical Requirements for Pharmaceuticals for Human Use (ICH) Q3A (R2) (International Council for Harmonisation of Technical Requirements for Pharmaceuticals for Human Use 2006), when the maximum daily dose exceeds 2 g/day, constituents with a content of ≥0.03% (0.3 mg/g) are subject to reporting. We therefore adopted this value as the cutoff for peak selection. Subsequently, each peak was then further analyzed *via* LC-MS to identify target ions. The 0.3 mg/g threshold was uniformly applied across all crude drugs. To calculate the content of each compound, peak intensities were compared to that of the glycyrrhizic acid target peak, and peak selection was performed automatically through computational processing to ensure consistency. Glycyrrhizic acid was chosen as the reference compound because it is a major, nonvolatile component found in many Kampo formulations, including HST.

*Component identification and estimation*

HST’s constituent crude drugs were analyzed *via* liquid chromatography time-of-flight mass spectrometry (LC-TOF-MS; Agilent 1,290 Infinity II LC, Agilent Technologies, CA, USA) coupled to a SCIEX TripleTOF 4600 mass spectrometer (SCIEX, MA, USA). Target components were estimated by comparing the MS spectra, exact masses, retention times, and base species information for each peak with data from the Dictionary of Natural Products. Component identities were confirmed *via* comparison with reference standards.

*Conditions for LC-MS analysis of HST extract*

LC analysis was performed on the Agilent Infinity 1,290 system equipped with a ZORBAX Eclipse Plus C18 column (100 × 2.1 mm, 1.8 μm) at 40 °C. Mobile phase A consisted of 0.1% formic acid in water; mobile phase B comprised acetonitrile:water:formic acid (100:10:0.1, v/v/v). Chromatographic separation was performed using the following gradient: 0.0–0.5 min, 5.0% B (isocratic; 0.6 mL/min); 0.5–10.0 min, 5.0%–30.0% B (linear gradient; 0.6 mL/min); 10.0–17.0 min, 30.0%–99.5% B (linear gradient; 0.6 mL/min); 17.0–18.5 min, 99.5% B (isocratic; 0.6–1.0 mL/min); 18.5–18.6 min, 99.5%–5.0% B (linear gradient; 1.0–0.6 mL/min); and 18.6–20.0 min, 5.0% B (isocratic; 0.6 mL/min). The injection volume was 2.0 μL. The LC system was coupled to an Agilent MSD XT 6135 mass analyzer *via* an electrospray ionization source, optimized with the following parameters: dry gas temperature, 350 °C; gas flow rate, 12.0 L/min; nebulizer pressure, 55 psi; capillary voltage, 3,500 V (positive/negative).

### Quantitative analysis

*Preparation of HST extract solution*

Three lots of HST powder (Lot# 372200900, 382092900, and 392165800) were provided by TSUMURA & CO. and used for quantitative analysis. Each HST powder (∼30 mg; actual range: 29.90–31.10 mg) was weighed into a 2 mL microcentrifuge tube, and 1.5 mL of 75% ethanol solution (ethanol:purified water = 3:1, v/v) was added. The mixture was shaken at 1,600 rpm for 30 min, followed by centrifugation at 2,000 × g for 3 min. The supernatant was then transferred to a glass vial and centrifuged at 7,000 rpm for 5 min to prepare the sample solution for quantitative analysis.

*Preparation of standard solutions*

Eight compounds, [6]-gingerol, [8]-gingerol, [10]-gingerol, [6]-shogaol, [8]-shogaol, [10]-shogaol, neoglycyrol, and glycycoumarin were used as reference standards for quantification. Each reference standard (∼0.5 mg; actual range: 0.45–0.55 mg) was weighed into a 10 mL volumetric flask, dissolved, and diluted to volume with 75% ethanol solution to prepare Standard Solution I. Subsequently, 1.58 mL of Standard Solution I was transferred into a 5 mL volumetric flask and diluted to volume with 75% ethanol to obtain Standard Solution II. This serial dilution process was repeated in the same manner to prepare Standard Solutions III, IV, and V, using 1.58 mL of the preceding solution for each step.

*Conditions for LC-MS analysis of HST extract solution and standard solutions*

LC-MS analysis was performed on the Agilent Infinity 1290 system equipped with a ZORBAX Eclipse Plus C18 column (100 × 2.1 mm, 1.8 μm) at 40 °C. Mobile phase A consisted of 0.1% formic acid in water; mobile phase B comprised acetonitrile:water:formic acid (100:10:0.1, v/v/v). Chromatographic separation was performed using the following gradient: 0.0–0.5 min, 5.0% B (isocratic; 0.6 mL/min); 0.5–10.0 min, 5.0%–30.0% B (linear gradient; 0.6 mL/min); 10.0–17.0 min, 30.0%–99.5% B (linear gradient; 0.6 mL/min); 17.0–18.5 min, 99.5% B (isocratic; 0.6–1.0 mL/min); 18.5–18.6 min, 99.5%–5.0% B (linear gradient; 1.0–0.6 mL/min); and 18.6–20.0 min, 5.0% B (isocratic; 0.6 mL/min). The injection volume was 2.0 μL. The LC system was coupled to an Agilent MSD XT 6135 mass analyzer *via* an electrospray ionization source, optimized with the following parameters: dry gas temperature, 350 °C; gas flow rate, 12.0 L/min; nebulizer pressure, 55 psi; capillary voltage, 3,500 V (positive/negative).

*Data analysis*

Eight reference standards were analyzed at five concentration levels (seven data points in total), consisting of Standard Solution I (50.0 µg/mL, injected at 0.3, 0.4, and 0.5 µL corresponding to 15.0, 20.0, and 25.0 ng, respectively), and Standard Solutions II–V at 16.0, 5.0, 1.6, and 0.5 µg/mL (each injected at 0.5 µL, corresponding to 8.0, 2.5, 0.8, and 0.25 ng, respectively). Calibration curves were generated using the weighted least-squares method (weights = reciprocal of the unbiased variance). Either a linear or quadratic regression model was applied, and the regression model showing the higher coefficient of determination (R^2^) was adopted. Before quantification, it was confirmed that the peak intensities of the HST extract samples (Lot# 372200900, 382092900, and 392165800) were within the upper and lower limits of the calibration curves. Subsequently, the contents of each marker compound in these lots were calculated.

### Statistical analysis

Data are presented as means ± standard deviations (SDs). IC_50_ values were calculated through fitting to a four-parameter logistic model using Prism 7 (GraphPad Software, CA, USA). Pearson’s correlation coefficients between pharmacological activity and multicomponent profiles, and analysis of variance of pharmacological activity of 101 HST samples were calculated using R statistical software (version 4.4.2; R Foundation for Statistical Computing, Vienna, Austria).

## Results

### Cell-based bioassay

To develop a cell-based assay for anti-inflammatory activity, cell density (0.5, 1.0, 2.0, and 4.0 × 10^4^ cells/well), assay duration (2, 6, and 24 h), and IL-1β concentration (1, 3, 10, and 30 ng/mL) were altered and PGE2 production measured (Figure S1). PGE2 production levels were dependent on three variables (Figures S1A, S1B), with the following optimal assay conditions selected: 4.0 × 10^4^ cells/well, 24 h of incubation, and 1 ng/mL IL-1β. DCF, a COX-1/2 inhibitor and representative anti-inflammatory agent, was used to validate the assay. DCF suppressed PGE2 production in a concentration-dependent manner (IC_50_ = 0.31 nM; Figure S1C), confirming that the assay could reliably detect anti-inflammatory activity. DCF (0.3 nM) was used as a positive control in subsequent experiments to validate the assay’s suitability.

To assess whether the assay could differentiate between Kampo extracts with varying compositions, we evaluated 128 differing formulations at 100 μg/mL (Figure S2 and Table S3). PGE2 inhibition varied widely, depending on the constituent crude drugs. Among the tested formulations, HST exhibited the strongest suppression of PGE2 production (6.49% of IL-1β control), demonstrating that the assay responded sensitively to compositional differences.

**Figure 1.**
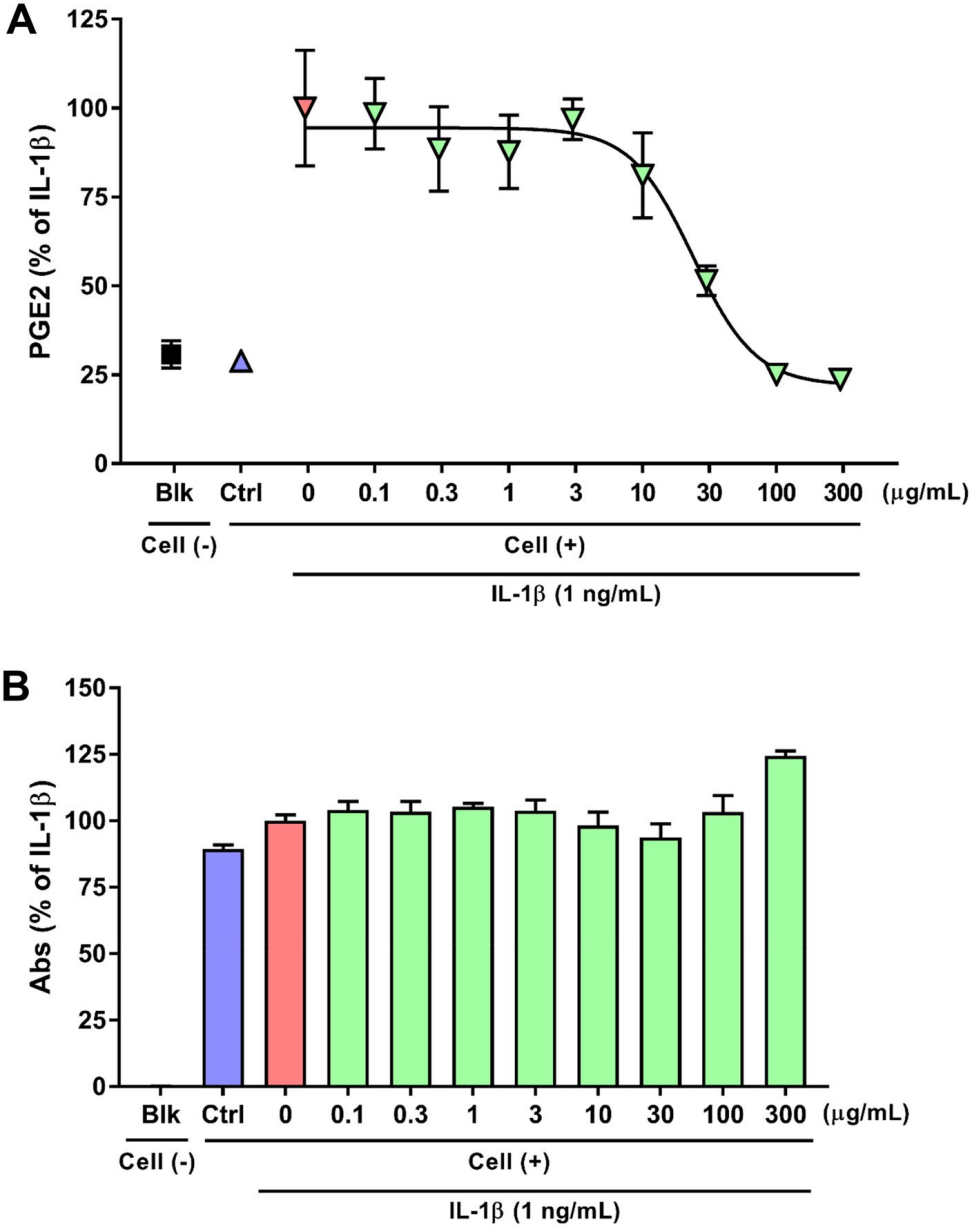
**(A)** Dose–response relationship between representative HST (Lot# 392107100) and 1 ng/mL IL-1β–induced PGE2 production in HOK cells (*n* = 3 wells; IC_50_ = 23.84 μg/mL). **(B)** Effect of HST on HOK cell viability (*n* = 3 wells). Black plots and bars: medium + medium; blue plots and bars: medium + medium; red plots and bars: 1 ng/mL IL-1β + medium; green plots and bars: 1 ng/mL IL-1β + 0.1–300 μg/mL HST. Blk: blank; ctrl: control. Data are means ± SDs (error bars).

Using the established cell-based assay, we tested a representative HST sample. HST inhibited PGE2 production in a concentration-dependent manner (IC_50_ = 23.84 μg/mL; Figure 1A). No cytotoxicity was observed at concentrations up to 300 μg/mL, as assessed by the effect of HST on HOK cells using the reduction of WST-8 as an indicator (Figure 1B). Furthermore, there is no influence of HST alone on cytotoxicity and PGE2 production in HOK cells (Figure S1D).

### Pharmacological activity and multicomponent analysis of 101 HST samples

**Figure 2.**
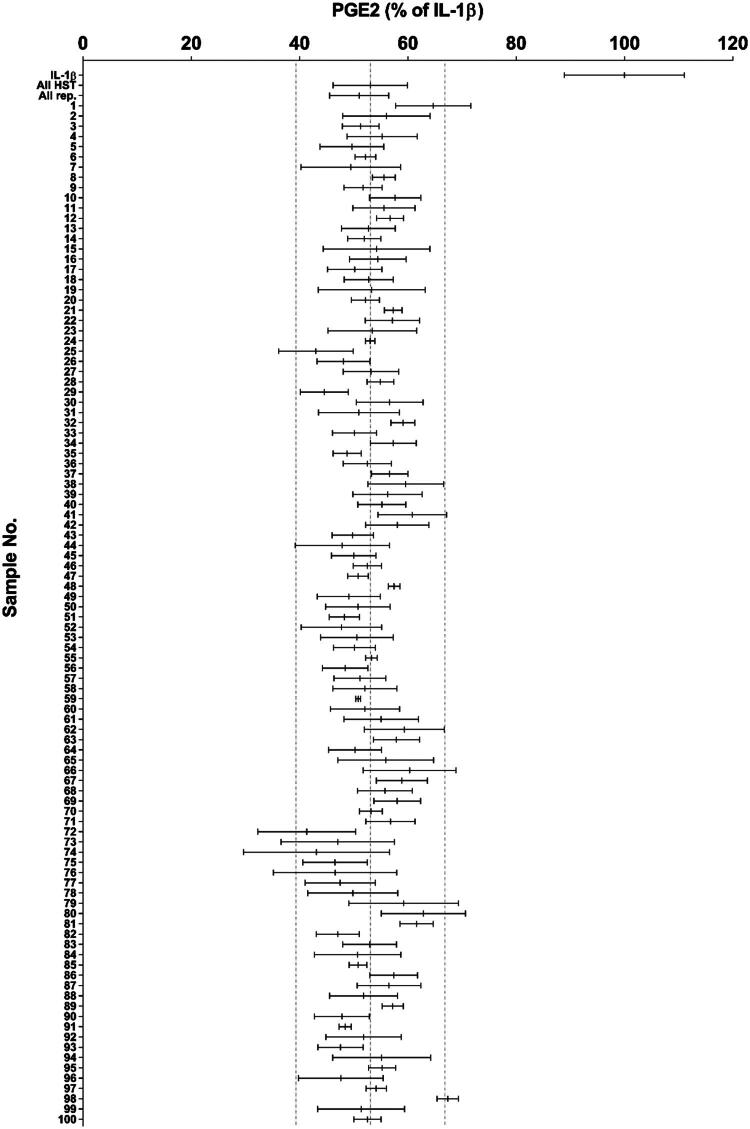
Comparison of 1 ng/mL IL-1β–induced PGE2 production among 101 HST samples (30 μg/mL) (IL-1β, *n* = 44 wells; All HST, *n* = 399 wells; All rep., *n* = 44 wells; each sample, *n* = 3–4 wells). All HST represents the average of 100 HST samples excluding the representative HST (rep., Lot# 392107100). All rep. represents the average of the representative HST across assay plates. All values were standardized using the representative HST. Dotted lines indicate the mean of All HST (53.08%) ± 2SD. Data are means ± SDs (error bars). HST: Hangeshashinto; rep.: representative sample.

The 101 tested HST samples were standardized for baicalin, berberine, and glycyrrhizic acid content, but other constituents may have varied among lots. Using a concentration near the IC_50_ (30 μg/mL; Figure 1A), we evaluated anti-inflammatory activity across all samples. All 101 lots inhibited PGE2 production with inhibition rates of 41.33%–67.38% (Figure 2 and Table 1), indicating consistent pharmacological efficacy. However, minor differences in inhibition rates were observed. Analysis of variance excluding the reference lot revealed that between-group variation significantly exceeded within-group variation [F (99, 299) = 2.58, *p* < 0.0001]. To identify the potential contributing components, we conducted multicomponent analysis.

**Table 1.** Inhibition ratio of PGE2 production (% of IL-1β) for 101 HST samples (*n* = 3–4).

Sample No.	PGE2 (%)	SD
IL-1β	100.00	11.08
All HST	53.08	4.63
All rep.	51.01	5.46
1	64.69	6.96
2	56.03	8.07
3	51.29	3.37
4	55.25	6.48
5	49.67	5.87
6	52.15	1.91
7	49.48	9.21
8	55.59	2.11
9	51.73	3.49
10	57.67	4.73
11	55.59	5.73
12	56.72	2.48
13	52.71	4.97
14	51.96	3.05
15	54.23	9.84
16	54.45	5.23
17	50.19	5.01
18	52.78	4.54
19	53.34	9.86
20	52.16	2.58
21	57.29	1.64
22	57.14	5.00
23	53.46	8.20
24	53.05	0.89
25	43.02	6.89
26	48.12	4.89
27	53.18	5.14
28	54.92	2.46
29	44.58	4.42
30	56.64	6.17
31	50.98	7.49
32	59.10	2.21
33	50.15	4.06
34	57.32	4.24
35	48.79	2.59
36	52.52	4.47
37	56.64	3.38
38	59.60	7.00
39	56.25	6.39
40	55.20	4.42
41	60.82	6.33
42	58.06	5.84
43	49.81	3.82
44	47.89	8.71
45	50.01	4.13
46	52.53	2.62
47	50.82	1.89
48	57.47	1.06
49	49.10	5.84
50	50.80	5.95
51	48.28	2.80
52	47.74	7.44
53	50.61	6.70
54	50.15	3.87
55	53.30	1.07
56	48.43	4.19
57	51.15	4.81
58	52.06	5.90
59	50.84	0.45
60	52.09	6.39
61	55.06	6.88
62	59.37	7.39
63	57.90	4.26
64	50.23	4.90
65	55.90	8.85
66	60.32	8.55
67	58.90	4.72
68	55.77	5.04
69	58.03	4.32
70	53.18	2.10
71	56.79	4.55
72	41.33	9.02
73	47.04	10.46
74	43.14	13.50
75	46.56	5.93
76	46.57	11.38
77	47.49	6.48
78	49.85	8.30
79	59.23	10.10
80	62.87	7.79
81	61.61	3.07
82	47.06	3.95
83	52.95	4.96
84	50.72	7.99
85	50.82	1.64
86	57.40	4.40
87	56.52	5.88
88	51.81	6.26
89	57.20	1.97
90	47.83	5.07
91	48.42	1.13
92	51.84	6.97
93	47.57	4.19
94	55.17	9.04
95	55.27	2.51
96	47.64	7.81
97	54.17	1.91
98	67.38	1.97
99	51.39	8.02
100	52.56	2.50

HST: Hangeshashinto; rep.: representative sample.

Each sample was analyzed using targeted LC-MS. In total, 121 peaks were selected based on LC-CAD profiling, and these peaks were subsequently analyzed using LC-TOF-MS for structural characterization. Of these, 55 were identified using authentic standards (based on exact mass, retention time, and MS pattern), and 66 were tentatively assigned using the Dictionary of Natural Products database. These 121 peaks were used as analytical targets for subsequent correlation analysis (Table 2).

**Table 2.** LC-MS analytical targets for HST samples.

Peak No.	t_R (min)_	Polarity	*m/z*	Fragmentor	Identification	CAS No.
1	1.27	Negative	719.2	150V		
2	2.82	Positive	107.1	150V		
3	3.95	Negative	367.1	150V		
4	4.61	Positive	257.1	150V	Glucoliquiritin	93446-18-5
5	4.71	Negative	303.2	150V	2,3,5,6,7-pentahydroxyflavanone	80366-15-0
6	4.94	Negative	549.4	150V		
7	5.12	Positive	342.3	150V	Magnoflorine	2141-09-5
8	5.14	Positive	219.2	150V		
9	5.31	Positive	595.4	150V	Vicenin II	23666-13-9
10	5.41	Positive	369.2	150V	3-O-Feruloylquinic acid	62929-69-5
11	6.08	Positive	565.4	150V	Schaftoside	51938-32-0
12	6.1	Positive	303.1	150V		
13	6.68	Negative	417.1	150V	Liquiritin	551-15-5
14	6.8	Negative	549.4	150V	Liquiritin apioside	74639-14-8
15	6.84	Positive	549.3	150V		
16	7.05	Positive	549.3	150V		
17	7.38	Positive	322.3	150V	Groenlandicine	38691-95-1
18	7.33	Positive	549.3	150V		
19	7.45	Positive	324.3	150V		
20	7.53	Positive	549.3	150V		
21	7.64	Positive	352.3	150V		
22	7.67	Negative	581.5	150V		
23	7.76	Positive	549.3	150V	Chrysin 6-C-glucoside 8-C-arabinoside	185145-34-0
24	7.83	Positive	549.3	150V		
25	8.04	Negative	433.3	150V	Choerospondin	81202-36-0
26	8.14	Negative	415.3	150V		
27	8.2	Negative	459.4	150V		
28	8.49	Positive	320.3	150V	Coptisine	3486-66-6
29	8.62	Positive	336.3	150V	Epiberberine	6873-09-2
30	8.78	Positive	338.3	150V		
31	8.78	Positive	477.2	150V		
32	9.04	Negative	549.4	150V	Isoliquiritin apioside	120926-46-7
33	8.95	Positive	289.1	150V		
34	9.03	Positive	347.4	150V		
35	9.24	Negative	417.1	150V	Isoliquiritin	5041-81-6
36	9.36	Positive	447.0	150V	Baicalin	21967-41-9
37	9.41	Negative	962.1	150V	20-O-Glucoginsenoside Rf	68406-27-9
38	9.55	Negative	255.1	150V	Liquiritigenin	578-86-9
39	9.7	Negative	932.0	150V	Notoginsenoside R1	80418-24-2
40	9.79	Positive	507.2	150V		
41	9.89	Positive	336.3	150V	Berberine	2086-83-1
42	9.97	Positive	352.3	150V		
43	10.02	Negative	695.6	150V		
44	10.12	Negative	891.7	150V		
45	10.23	Negative	946.1	150V	Ginsenoside Re	52286-59-6
46	10.23	Negative	845.3	150V	Ginsenoside Rg1	22427-39-0
47	10.42	Negative	299.1	150V		
48	10.52	Negative	269.1	150V		
49	10.62	Negative	859.7	150V	Chrysin-7-O-Glucuronide	35775-49-6
50	10.74	Positive	461.2	150V	Oroxyloside	36948-76-2
51	10.77	Negative	842.0	150V		
52	10.87	Positive	517.3	150V		
53	10.96	Negative	445.3	150V		
54	11.17	Positive	461.2	150V	Wogonoside	51059-44-0
55	11.39	Negative	823.9	150V		
56	11.47	Positive	491.2	150V		
57	11.66	Negative	1000.0	150V		
58	11.81	Negative	895.9	150V		
59	11.85	Negative	821.8	150V		
60	11.97	Positive	271.0	150V		
61	12.06	Negative	984.0	150V	Licoricesaponin A3	118325-22-7
62	12.15	Negative	799.9	150V	Ginsenoside A1	69884-00-0
63	12.24	Negative	879.9	150V		
64	12.25	Positive	271.0	150V	Baicalein	491-67-8
65	12.33	Negative	837.8	150V		
66	12.38	Negative	1028.1	150V		
67	12.43	Negative	1107.4	150V	Ginsenoside Rb1	41753-43-9
68	12.43	Negative	576.8	150V		
69	12.49	Negative	1150.2	150V		
70	12.47	Negative	431.4	150V		
71	12.49	Positive	257.1	150V	Isoliquiritigenin	961-29-5
72	12.56	Negative	1078.2	150V	Ginsenoside Rc	11021-14-0
73	12.54	Positive	369.2	150V		
74	12.61	Negative	956.1	150V	Ginsenoside Ro	34367-04-9
75	12.68	Negative	1078.2	150V	Ginsenoside Rb2	11021-13-9
76	12.68	Negative	819.2	150V		
77	12.69	Negative	837.8	150V	Licoricesaponin G2	118441-84-2
78	12.73	Negative	1120.2	150V		
79	12.81	Negative	864.0	150V		
80	12.99	Negative	821.2	150V	Glycyrrhizic acid	1405-86-3
81	12.96	Negative	946.1	150V	Ginsenoside Rd	52705-93-8
82	13.03	Negative	988.1	150V		
83	13.24	Negative	807.9	150V		
84	13.34	Positive	261.3	150V		
85	13.39	Negative	821.8	150V		
86	13.38	Positive	285.0	150V	Wogonin	632-85-9
87	13.48	Negative	823.9	150V		
88	13.52	Positive	315.1	150V		
89	13.57	Positive	277.1	150V	[6]-Gingerol	23513-14-6
90	13.58	Negative	373.2	150V	Skullcapflavone II	55084-08-7
91	13.64	Negative	805.9	150V		
92	13.8	Positive	369.2	150V	Glycycoumarin	94805-82-0
93	13.91	Positive	353.2	150V		
94	14.03	Negative	353.3	150V		
95	14.14	Positive	341.3	150V		
96	14.3	Positive	367.3	150V	Neoglycyrol	23013-84-5
97	14.45	Positive	385.2	150V		
98	14.57	Negative	371.3	150V		
99	14.77	Positive	425.4	150V		
100	14.8	Positive	305.4	150V	[8]-Gingerol	23513-08-8
101	14.87	Positive	383.3	150V		
102	14.96	Negative	485.6	150V	Ceanothic acid	21302-79-4
103	15.28	Positive	277.1	150V	[6]-Shogaol	555-66-8
104	15.1	Negative	369.3	150V	Glyasperin D	142561-10-2
105	15.28	Positive	425.4	150V		
106	15.46	Positive	398.5	150V	Diacetoxy-6-gingerdiol	143615-75-2
107	15.59	Negative	353.3	150V		
108	15.64	Negative	421.4	150V	Isoangustone A	129280-34-8
109	15.77	Positive	421.3	150V		
110	15.81	Positive	423.3	150V		
111	15.93	Positive	333.5	150V	[10]-Gingerol	23513-15-7
112	15.99	Positive	471.5	150V	18b-Glycyrrhetinic acid	471-53-4
113	16.19	Positive	305.4	150V	[8]-Shogaol	36700-45-5
114	16.25	Positive	439.5	150V		
115	17.53	Positive	331.4	150V		
116	17.25	Positive	333.5	150V	[10]-Shogaol	36752-54-2
117	17.25	Positive	439.5	150V	Betulinic acid	472-15-1
118	17.47	Negative	253.2	150V	Palmitoleic acid	373-49-9
119	17.61	Positive	347.4	150V		
120	17.68	Negative	279.3	150V	Linoleic acid	60-33-3
121	17.87	Negative	453.5	150V	Oleanonic acid	17990-42-0

### Identification of active ingredients via correlation analysis

To identify candidate active ingredients, we performed correlation analysis between PGE2 inhibition data (Figure 2) and the peak intensities of the 121 components across the 101 HST samples. Correlation coefficients ranged from −0.45 to 0.40 (Figure 3A). Table 3 lists the top 20 ingredients with correlation coefficients below −0.30. Among these, 11 ingredients (schaftoside, 2-acetoxy-3-methoxybenzoic acid, glycycoumarin, neoglycyrol, chrysin 6-C-glucoside 8-C-arabinoside, magnoflorine, [6]-shogaol, [8]-shogaol, [10]-shogaol, [8]-gingerol, and [10]-gingerol) were commercially available and selected for validation. Additionally, baicalin, its aglycon baicalein, and [6]-gingerol (a structural analog of [6]-shogaol) were tested. Eight ingredients, namely glycycoumarin, neoglycyrol, [6]-shogaol, [8]-shogaol, [10]-shogaol, [6]-gingerol, [8]-gingerol, and [10]-gingerol, exhibited concentration-dependent inhibition of PGE2 production (Figure 3B).

Figure 3.**(A)** Pearson correlation between inhibition of PGE2 production and LC-MS peak intensity across 101 HST samples. Solid line indicates correlation coefficient = 0. **(B)** Effects of selected ingredients on 1 ng/mL IL-1β–induced PGE2 production in HOK cells (IL-1β, *n* = 32 wells; All DCF, *n* = 32 wells; each sample, *n* = 3–4 wells). All values were standardized using DCF (0.3 nM). Data are means ± SDs (error bars). DCF: diclofenac sodium; SFT: schaftoside; AMB: 2-acetoxy-3-methoxybenzoic acid; GLC: glycycoumarin; CGA: chrysin-6-C-glucoside 8-C-arabinoside; NGR: neoglycyrol; BCLi: baicalin; BCLe: baicalein; MGF: magnoflorine; [6]SG: [6]-shogaol; [8]SG: [8]-shogaol; [10]SG: [10]-shogaol; [6]GG: [6]-gingerol; [8]GG: [8]-gingerol; [10]GG: [10]-gingerol.

**Figure:**
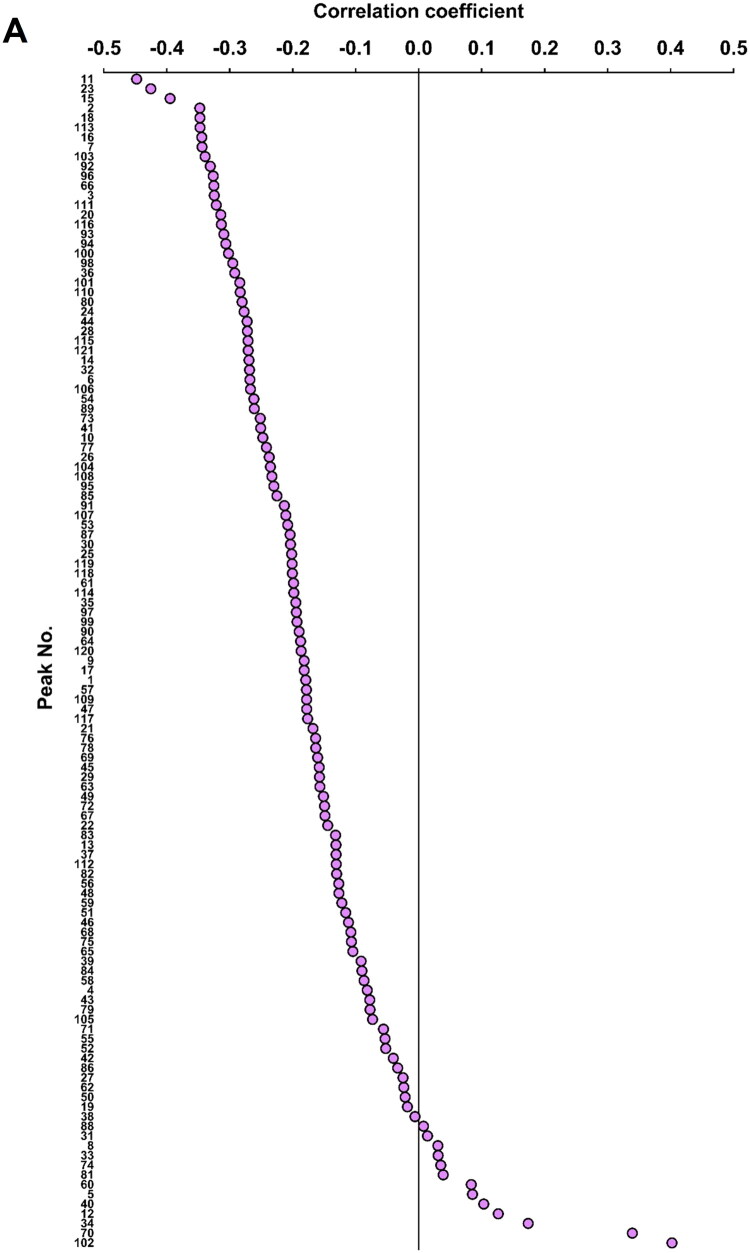

**Figure:**
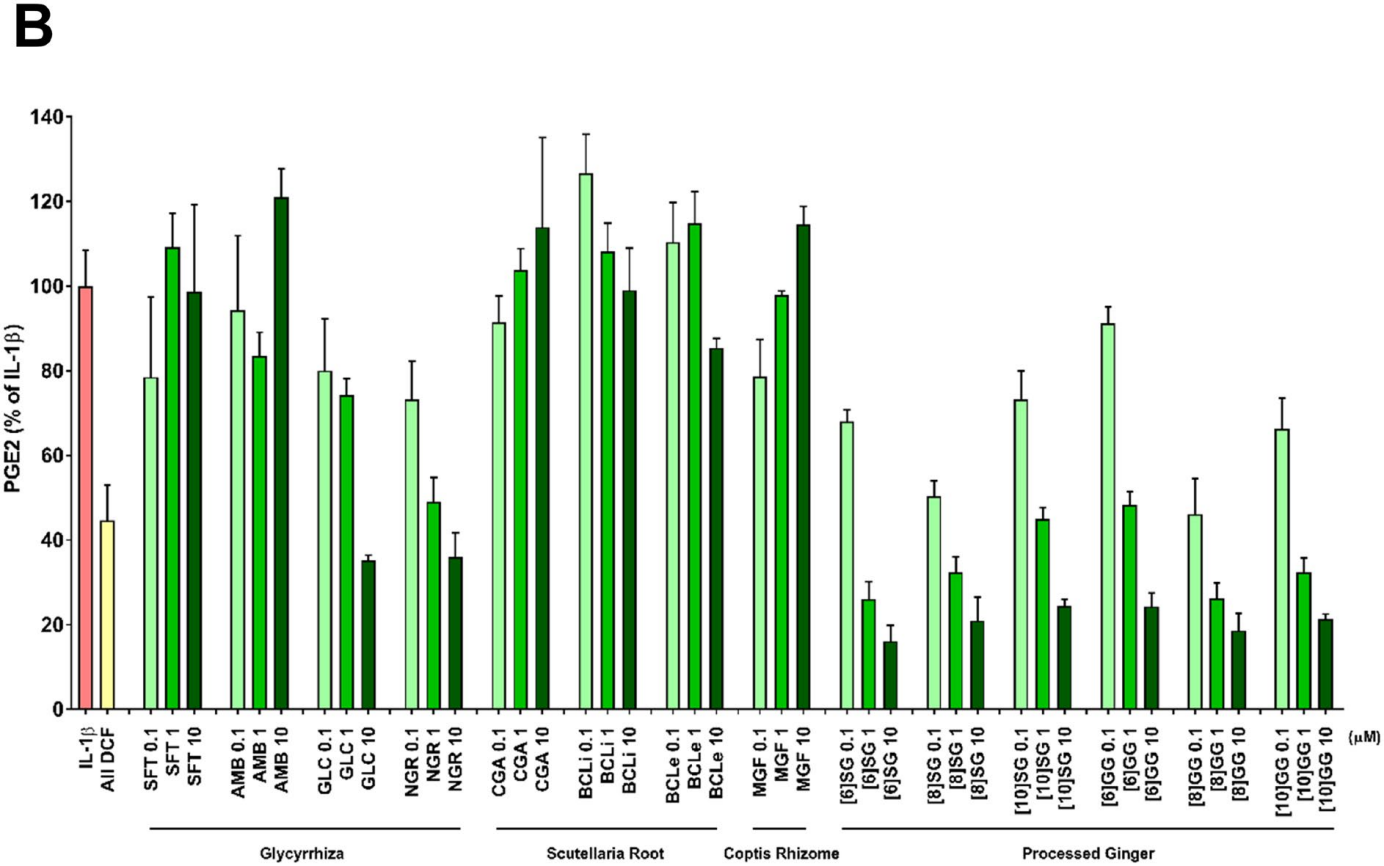

**Table 3.** List of peak numbers and correlation coefficients between PGE2 inhibition and LC-MS peak intensity across 101 HST samples.

Peak no.	Identification	Compound name	Correlation coefficient	*p*-value
11	IC	Schaftoside	−0.45	2.63 × 10^-6^
23	IC	Chrysin 6-C-glucoside 8-C-arabinoside	−0.43	9.22 × 10^-6^
15	EC	Chrysin 6-C-glucoside 8-C-arabinoside (isomer1)	−0.39	4.41 × 10^-5^
2	EC	2-acetoxy-3-methoxybenzoic acid	−0.35	3.68 × 10^-4^
7	IC	Magnoflorine	−0.35	3.72 × 10^-4^
113	IC	[8]-Shogaol	−0.35	3.73 × 10^-4^
18	EC	Chrysin 6-C-glucoside 8-C-arabinoside (isomer3)	−0.34	4.20 × 10^-4^
16	EC	Chrysin 6-C-glucoside 8-C-arabinoside (isomer2)	−0.34	4.27 × 10^-4^
103	IC	[6]-Shogaol	−0.34	5.20 × 10^-4^
66	EC	Hydroxy-licoricesaponin D3	−0.33	7.22 × 10^-4^
92	IC	Glycycoumarin	−0.33	8.68 × 10^-4^
111	IC	[10]-Gingerol	−0.33	9.07 × 10^-4^
96	IC	Neoglycyrol	−0.32	9.28 × 10^-4^
3	EC	1-O-Feruloylquinic acid	−0.32	1.04 × 10^-3^
116	IC	[10]-Shogaol	−0.31	1.37 × 10^-3^
100	IC	[8]-Gingerol	−0.31	1.42 × 10^-3^
94	EC	Gancaonin C	−0.31	1.64 × 10^-3^
20	EC	Chrysin 6-C-glucoside 8-C-arabinoside (isomer4)	−0.31	1.84 × 10^-3^
93	EC	Gancaonin A	−0.30	2.15 × 10^-3^
98	EC	Licoriphenone	−0.30	2.72 × 10^-3^

IC: identified compound; EC: estimated compound.

We performed quantitative analysis on three lots (372200900, 382092900, 392165800) of HST with different manufacturing years. Quantitative analysis of the eight components revealed the approximate content of each ingredient contained in HST (Table S4).

## Discussion

We established a cell-based assay to directly evaluate the anti-inflammatory activity of HST against stomatitis (Figures 1 and S1) and demonstrated that all 101 HST samples, controlled for baicalin, berberine, and glycyrrhizic acid as quality control markers, exhibited stable and expected anti-inflammatory activity (Figure 2 and Table 1). Although Kampo medicines are multicomponent formulations and may undergo metabolic transformation *in vivo*, our use of a cell-based assay was justified by the clinical application of HST as a gargle for treating chemotherapy-induced stomatitis, where it likely acts locally (Matsuda et al. 2015). Furthermore, the results of the analysis of variance indicated that the between-group variation was greater than the within-group variation, demonstrating sufficient power to detect differences in effect. This suggests that our bioassay method is sufficiently stable. We consider that this cell-based bioassay is a stable quality evaluation method. Thus, direct exposure of HOK cells to HST in this assay closely matches the clinical context and serves as a reasonable method for stable assessing its anti-inflammatory effects.

Although baicalin, berberine and glycyrrhizic acid are established as quality control markers for HST (Ministry of Health, Labour and Welfare 2021), other ingredients may vary among production lots. In this study, we tested 101 quality-controlled HST samples at 30 μg/mL (approximately the IC_50_; Figure 2 and Table 1) and observed inhibition of PGE2 production ranging from 41.33% to 67.38%. These results suggest that the current quality control markers adequately maintain pharmacological consistency. On the other hand, considering the results of the analysis of variance, it is also possible to interpret that slight differences in activity were observed between each lot. Given the observed range in inhibitory activity, we hypothesized that variability in ingredient composition among lots accounts for these differences. Therefore, we conducted a correlation analysis between the intensities of 121 LC-MS peaks and the PGE_2_ inhibition rates of 101 samples (Figure 3A). Unlike traditional methods that rely on selecting abundant or unique compounds in Kampo medicine, our method enabled a comprehensive, data-driven exploration for bioactive ingredients. Similar integrated strategies combining bioassays and chemical profiling to identify active ingredients have been reported previously (Li et al. 2019; Ma et al. 2021; Li et al. 2023; Harada et al. 2025), enabling unbiased identification of pharmacologically relevant constituents.

We performed a multicomponent intensity analysis to explore potential active ingredients (Figure 3A and Table 3), identifying eight ingredients (glycycoumarin, neoglycyrol, [6]-shogaol, [8]-shogaol, [10]-shogaol, [6]-gingerol, [8]-gingerol, and [10]-gingerol) as contributors to HST’s stomatitis-mitigating effects and potential pharmacological quality control markers (Figure 3B). Glycycoumarin and neoglycyrol have coumarin as their basic skeleton, while [6]-shogaol, [8]-shogaol, [10]-shogaol, [6]-gingerol, [8]-gingerol, and [10]-gingerol possess a phenylpropanoid skeleton. It is widely recognized that both skeletons exhibit anti-inflammatory effects in natural products (de Cassia da Silveira et al. 2014; Rostom et al. 2022), and a valid skeletal group has been identified. Specifically, glycycoumarin and neoglycyrol have previously been reported to inhibit PGE2 production in RAW264.7 macrophages (Shin et al. 2008; Fu et al. 2013; Tang et al. 2023), and [6]-shogaol, [8]-shogaol, [10]-shogaol, [6]-gingerol, [8]-gingerol, and [10]-gingerol have been shown to inhibit PGE2 production in HOK cells (Kono et al. 2014). Glycycoumarin may activate the Nrf2 antioxidant pathway, indirectly causing anti-inflammatory effects (Zang 2020; Zhang et al. 2020; Tang et al. 2023), whereas neoglycyrol, shogaols, and gingerols are known to suppress PGE2 expression *via* the COX-2 and NF-κB pathways (Shin et al. 2008; Mao et al. 2019; Bischoff-Kont and Furst 2021; Ballester et al. 2022; Ishfaq et al. 2022; Promdam and Panichayupakaranant 2022; Yücel et al. 2022; Sharma et al. 2023). These mechanisms align with the compounds’ strong negative correlations with PGE2 production in our analysis. As described above, while glycycoumarin and neoglycyrol are known to be anti-inflammatory substances, this study is the first to report their contribution to the anti-inflammatory effects of HST. We believe that selecting pharmacologically relevant quality control marker components in multi-component pharmaceuticals requires the following research and development process: 1) Identifying the primary active ingredients involved in specific pharmacological actions, 2) Manufacturing Kampo medicines that stably control these components, and 3) Validating the pharmacological activity and efficacy of these manufactured products through basic and clinical research. Repeating this cycle enables the selection of more convincing quality control marker components. This research corresponds to Process 1). The newly discovered anti-inflammatory ingredients glycycoumarin and neoglycyrol in this study are expected to advance not only to research on single-component research focusing on the anti-inflammatory effects of HST, but also to discuss the selection of pharmacological quality control markers in HST manufacturing.

In this study, a correlation coefficient of −0.3 was set as the criterion for correlation analysis in active ingredient exploration (Figure 3A and Table 3). This is because the criterion contains many HST ingredients ([6]-shogaol, [8]-shogaol, [10]-shogaol, [8]-gingerol, and [10]-gingerol), which have already been reported to possess PGE2 inhibitory activity (Kono et al. 2014). Furthermore, a correlation coefficient of ±0.3 is interpreted as indicating a weak but significant correlation (Mukaka 2012; Akoglu 2018; Schober et al. 2018). For the above two reasons, this study defined active ingredients as those with a correlation coefficient of −0.3 or lower. The 101 lots of HST samples used in this study were manufactured using crude drugs conforming to the Japanese Pharmacopeia and quality-controlled based on the quality markers (baicalin, berberine, and glycyrrhizic acid). Even within such a homogeneous sample set, the eight active ingredients identified (glycycoumarin, neoglycyrol, [6]-shogaol, [8]-shogaol, [10]-shogaol, [6]-gingerol, [8]-gingerol, and [10]-gingerol) are considered promising candidates for pharmacological quality control markers. On the other hand, only 8 of the 11 ingredients (excluding the 3 arbitrarily added components) whose activity was verified possessed activity, and the detection rate of active ingredients fell short of expectations. In a sample set with low diversity, the active ingredients detected by correlation analysis may have been limited. To detect more convincing active ingredients, it may be necessary to conduct analyses using diverse sample sets and to explore modified correlation analysis methods.

Based on the quantitative analysis (Table S4), the total content of 8 ingredients at 30 μg/mL HST (approximately the IC_50_) is around 0.1 µM. Their mean individual activity at this concentration, 68.56%, accounted for ∼63% of the observed HST activity. This suggests that stable control of these eight ingredients, in addition to the currently monitored markers, could enhance the anti-inflammatory quality of HST. Previous studies have reported that baicalin, berberine, and wogonin also possess anti-inflammatory properties (Kono et al. 2014), and these, along with other unidentified ingredients, may contribute to the remaining activity. However, incorporating low-abundance compounds as quality markers carries the risk of variability, as such ingredients can be easily lost or may be difficult to detect and quantify reliably. Therefore, it is desirable to select additional markers among the identified active ingredients that are technically feasible to measure and capable of improving the consistency of HST’s anti-inflammatory effects.

## Conclusions

We demonstrated that consistent anti-inflammatory effects can be expected using the current quality control markers. Conversely, the addition of eight newly identified ingredients as quality control markers may further stabilize the anti-inflammatory effects. Furthermore, the combination of cell-based bioassays and multicomponent analysis is beneficial for objective screening of active ingredients.

We acknowledge that the current findings alone cannot fully explain clinical efficacy or predict clinical outcomes. Validation of the pharmacological effects of the eight identified ingredients using animal models with stomatitis and multicomponent analysis in HST lots that have shown stomatitis-improving effects in clinical practice are further required. Nevertheless, the correlation between the cell-based assay data and multicomponent profiles offers a promising foundation for future research. With further validation, this approach could support the development of predictive *in vitro* assays for evaluating the clinical efficacy of multicomponent drugs, including Kampo formulations.

## Supplementary Material

Supplemental Material

Supplemental materials_Crude drug list for 128 Kampo formulas.xlsx

## Data Availability

The authors confirm that the data supporting the findings of this study are available within the article and its supplementary material.

## Supplemental materials

Detailed background data for establishing the cell-based assay system and quantitative analysis data for eight ingredients (docx). List of crude drugs for 128 Kampo formulas (xlsx).
