# Supplementary material for: Potential pharmacological quality control markers in the traditional Japanese medicine Hangeshashinto: identifying anti-inflammatory ingredients through a cell-based bioassay and multicomponent analysis
Source: Pharm Biol. 2025 Nov 15;63(1):819–36. doi: 10.1080/13880209.2025.2583068 (PMC12621343; doi:10.1080/13880209.2025.2583068)
Supplement: Supplemental Material [file IPHB_A_2583068_SM0665.docx]

**Potential pharmacological quality control markers in the traditional Japanese medicine Hangeshashinto: Identifying anti-inflammatory ingredients through a cell-based bioassay and multicomponent analysis**

Ryota Imai ^*^, Yuta Muraki, Akinori Nishi, Katsuya Ohbuchi

TSUMURA Advanced Technology Research Laboratories, Research & Development Division, TSUMURA & CO., Ibaraki, Japan

^*^Correspondence: Ryota Imai Ph. D.

E-mail: imai_ryouta@mail.tsumura.co.jp

Telephone: +81(0)29-889-3844

Fax: +81(0)29-889-3870

3586 Yoshiwara, Ami-machi, Inashiki-gun, Ibaraki Postal#300-1192, Japan

**Table S1.** Lot numbers of HST samples used in this study.

| **Sample no.** | **Lot no.** | **Sample no.** | **Lot no.** | **Sample no.** | **Lot no.** | **Sample no.** | **Lot no.** |
| --- | --- | --- | --- | --- | --- | --- | --- |
| 1 | 382027200 | 26 | 382092600 | 51 | 382182200 | 76 | 392166400 |
| 2 | 382027300 | 27 | 382092800 | 52 | 392106700 | 77 | 392166500 |
| 3 | 382027100 | 28 | 382182300 | 53 | 392106800 | 78 | 392166600 |
| 4 | 382027000 | 29 | 382182400 | 54 | 392106900 | 79 | 392182900 |
| 5 | 382026900 | 30 | 382182500 | 55 | 392107000 | 80 | 392183000 |
| 6 | 382026700 | 31 | 382182600 | 56 | 392165400 | 81 | 392183100 |
| 7 | 382026600 | 32 | 382182700 | 57 | 392165500 | 82 | 382093100 |
| 8 | 382027400 | 33 | 382182800 | 58 | 392165600 | 83 | 382093200 |
| 9 | 382026500 | 34 | 382182900 | 59 | 392165700 | 84 | 382093300 |
| 10 | 382026400 | 35 | 392029200 | 60 | 392165800 | 85 | 382093400 |
| 11 | 382026300 | 36 | 392029300 | 61 | 392165900 | 86 | 382093500 |
| 12 | 382026200 | 37 | 392029400 | 62 | 392183200 | 87 | 382093600 |
| 13 | 372201900 | 38 | 392029500 | 63 | 392183300 | 88 | 382093700 |
| 14 | 372201800 | 39 | 392029600 | 64 | 392183400 | 89 | 382093800 |
| 15 | 372201700 | 40 | 392029700 | 65 | 392183500 | 90 | 392105900 |
| 16 | 372201600 | 41 | 392029800 | 66 | 392183600 | 91 | 392106000 |
| 17 | 372201500 | 42 | 392029900 | 67 | 392183700 | 92 | 392106100 |
| 18 | 372201400 | 43 | 392030000 | 68 | 392183800 | 93 | 392106200 |
| 19 | 372201300 | 44 | 392030100 | 69 | 392183900 | 94 | 392106300 |
| 20 | 372201200 | 45 | 392030200 | 70 | 392184000 | 95 | 392106400 |
| 21 | 372201100 | 46 | 392030400 | 71 | 392184100 | 96 | 392106500 |
| 22 | 372201000 | 47 | 382181700 | 72 | 392166000 | 97 | 392106600 |
| 23 | 372200900 | 48 | 382181900 | 73 | 392166100 | 98 | 372200800 |
| 24 | 382092900 | 49 | 382182000 | 74 | 392166200 | 99 | 382026800 |
| 25 | 382092700 | 50 | 382182100 | 75 | 392166300 | 100 | 382093000 |
|  |  |  |  |  |  | rep. | 392107100 |

Rep.: Representative sample.

**Table S2.** Lot numbers of Kampo samples in this study.

| **Kampo no.** | **Lot no.** | **Kampo no.** | **Lot no.** | **Kampo no.** | **Lot no.** | **Kampo no.** | **Lot no.** |
| --- | --- | --- | --- | --- | --- | --- | --- |
| TJ-1 | 411111000 | TJ-35 | 411115300 | TJ-70 | 411056100 | TJ-103 | 411090800 |
| TJ-2 | 392138900 | TJ-36 | 411082300 | TJ-71 | 411105600 | TJ-104 | 411097000 |
| TJ-3 | 411114300 | TJ-37 | 411117800 | TJ-72 | 391244900 | TJ-105 | 411101900 |
| TJ-5 | 411064700 | TJ-38 | 402004900 | TJ-73 | 411093700 | TJ-106 | 392187000 |
| TJ-6 | 392207800 | TJ-39 | 392080800 | TJ-74 | 381215000 | TJ-107 | 411117300 |
| TJ-7 | 411136500 | TJ-40 | 392210200 | TJ-75 | 391220500 | TJ-108 | 392210000 |
| TJ-8 | 392169500 | TJ-41 | 402012400 | TJ-76 | 411137800 | TJ-109 | 392150000 |
| TJ-9 | 392155000 | TJ-43 | 402005800 | TJ-77 | 411030700 | TJ-110 | 411100500 |
| TJ-10 | 392182200 | TJ-45 | 391145900 | TJ-78 | 391242200 | TJ-111 | 411132500 |
| TJ-11 | 411088400 | TJ-46 | 401033100 | TJ-79 | 391161100 | TJ-112 | 411123900 |
| TJ-12 | 392206800 | TJ-47 | 412138300 | TJ-80 | 411110100 | TJ-113 | 411045900 |
| TJ-14 | 392106500 | TJ-48 | 392180100 | TJ-81 | 391032500 | TJ-114 | 392133100 |
| TJ-15 | 392125000 | TJ-50 | 392187200 | TJ-82 | 411105900 | TJ-115 | 411037400 |
| TJ-16 | 411122600 | TJ-51 | 392198400 | TJ-83 | 412135600 | TJ-116 | 411107900 |
| TJ-17 | 411125100 | TJ-52 | 391247400 | TJ-84 | 392112400 | TJ-117 | 411104200 |
| TJ-18 | 402000100 | TJ-53 | 411113400 | TJ-85 | 391230600 | TJ-118 | 411085400 |
| TJ-19 | 402006900 | TJ-54 | 412144800 | TJ-86 | 392205200 | TJ-119 | 411134600 |
| TJ-20 | 392002500 | TJ-55 | 411113500 | TJ-87 | 392079800 | TJ-120 | 381001800 |
| TJ-21 | 411065100 | TJ-56 | 411062300 | TJ-88 | 411103100 | TJ-121 | 391021300 |
| TJ-22 | 411050600 | TJ-57 | 392109900 | TJ-89 | 411119000 | TJ-122 | 401018600 |
| TJ-23 | 392061800 | TJ-58 | 411122800 | TJ-90 | 411131100 | TJ-123 | 401018400 |
| TJ-24 | 411128900 | TJ-59 | 391093500 | TJ-91 | 411132600 | TJ-124 | 411127900 |
| TJ-25 | 402009700 | TJ-60 | 392201000 | TJ-92 | 411035700 | TJ-125 | 392134600 |
| TJ-26 | 411139900 | TJ-61 | 392195400 | TJ-93 | 391185800 | TJ-126 | 402006120 |
| TJ-27 | 391254300 | TJ-62 | 411118900 | TJ-95 | 411081500 | TJ-127 | 411141900 |
| TJ-28 | 401025000 | TJ-63 | 392207700 | TJ-96 | 392164700 | TJ-128 | 391068800 |
| TJ-29 | 3911899001 | TJ-64 | 411037100 | TJ-97 | 391174600 | TJ-133 | 411055600 |
| TJ-30 | 411137300 | TJ-65 | 401018700 | TJ-98 | 401019800 | TJ-134 | 392203400 |
| TJ-31 | 411131600 | TJ-66 | 391133800 | TJ-99 | 392136000 | TJ-135 | 371208000 |
| TJ-32 | 411054700 | TJ-67 | 411045400 | TJ-100 | 392205100 | TJ-136 | 392207300 |
| TJ-33 | 411072700 | TJ-68 | 411135500 | TJ-101 | 381066900 | TJ-137 | 392210900 |
| TJ-34 | 411142300 | TJ-69 | 411055000 | TJ-102 | 411099900 | TJ-138 | 411112300 |

**Figure S1.**


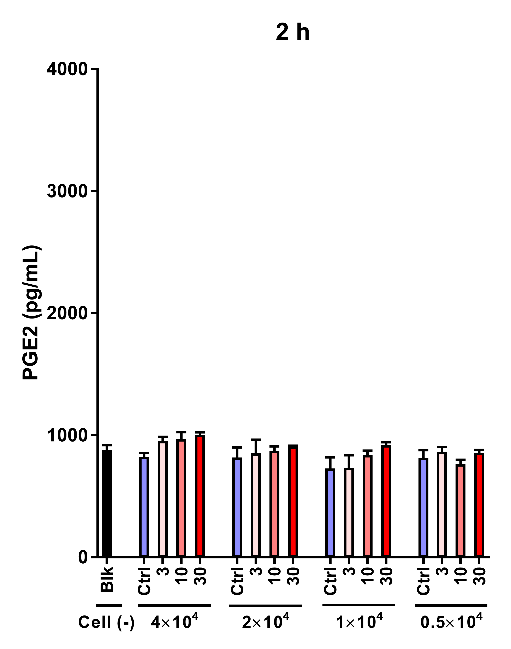

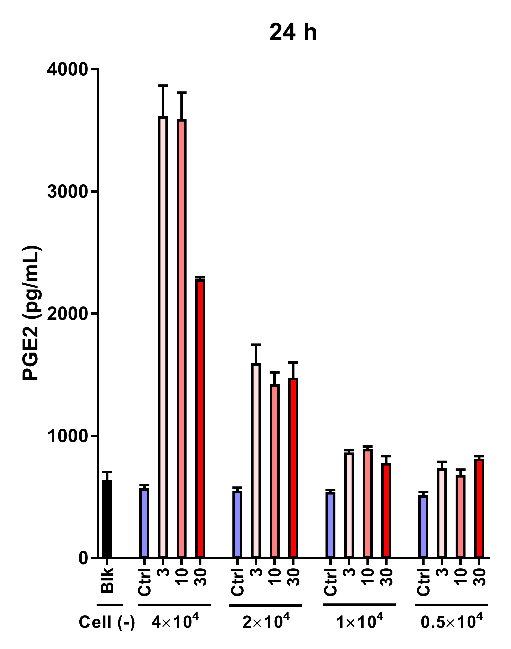


**A**


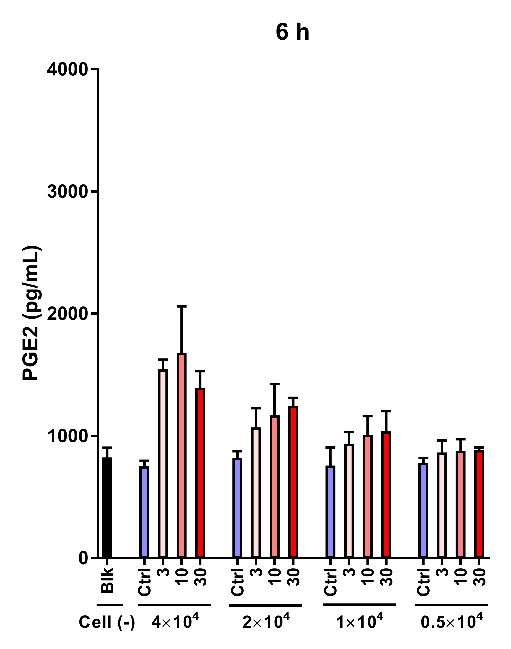


**B**


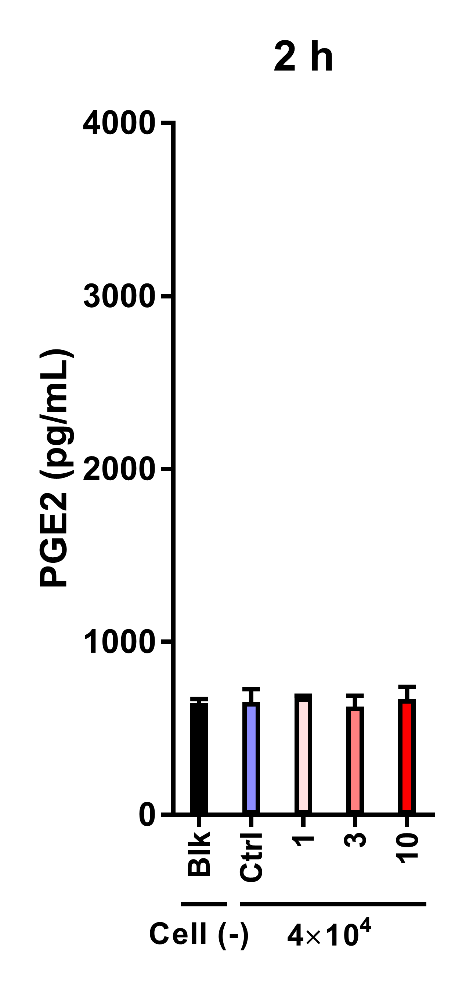

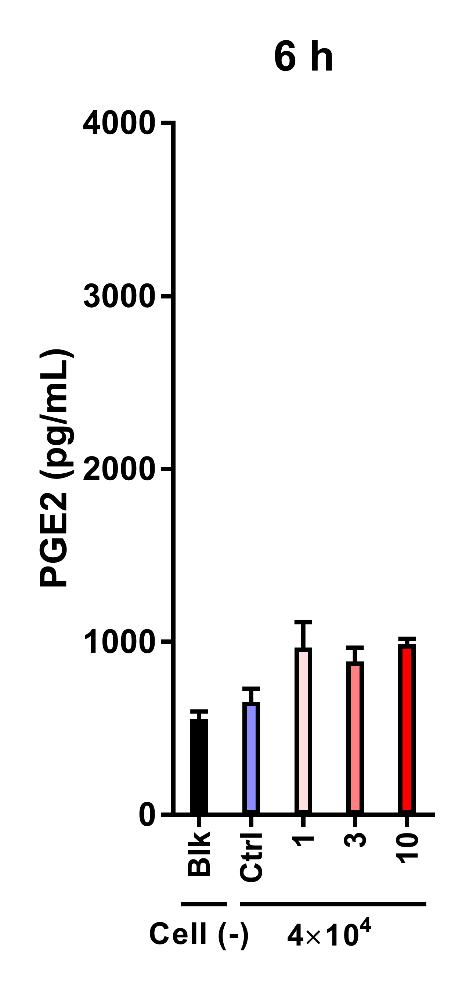

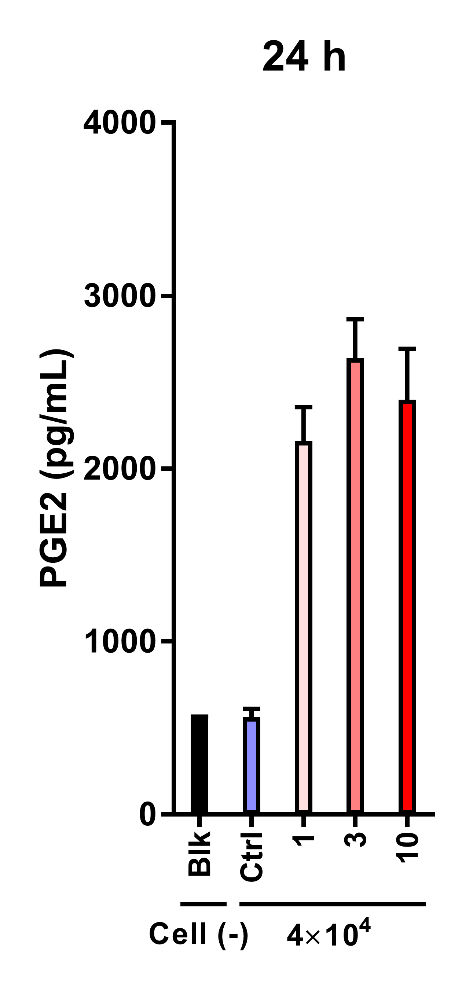


**C**


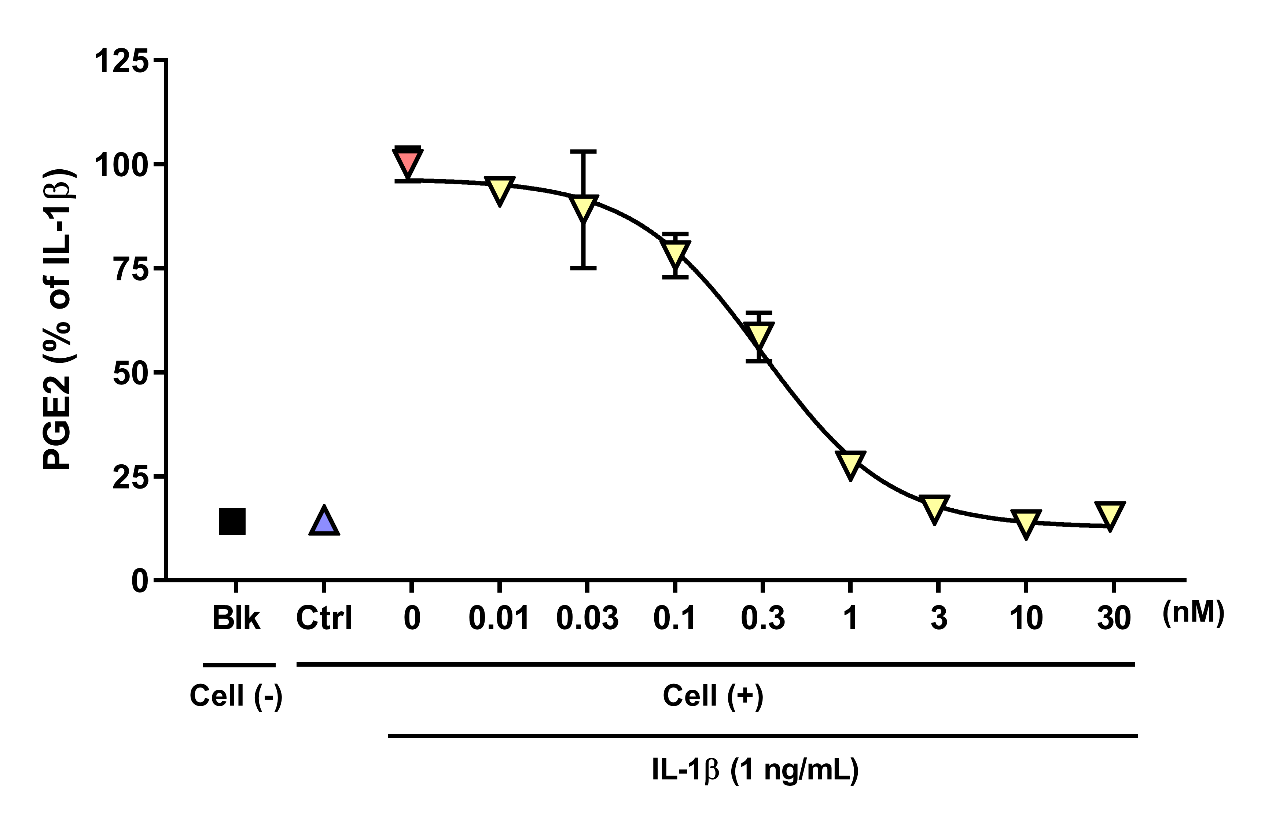


**D**


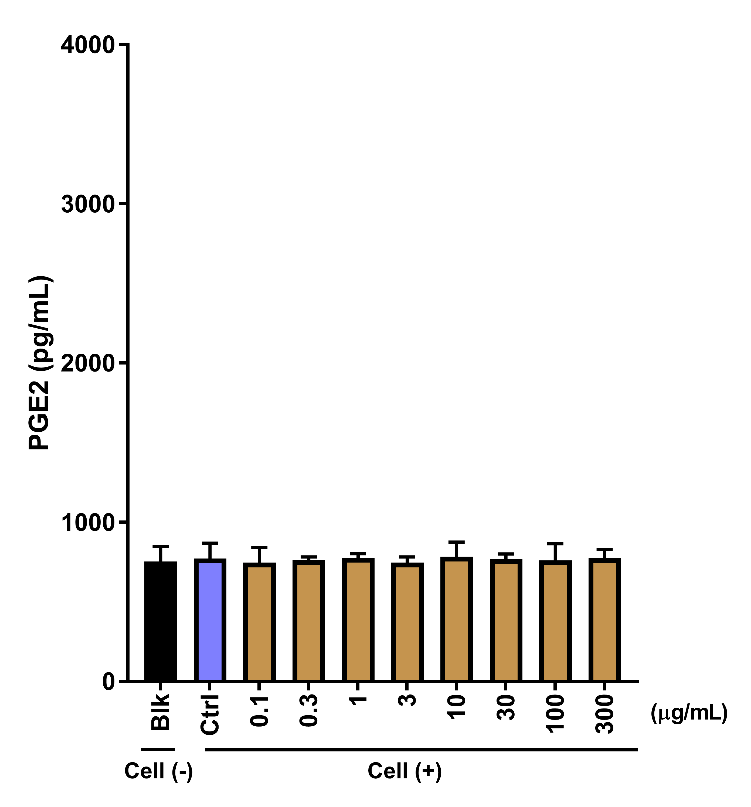

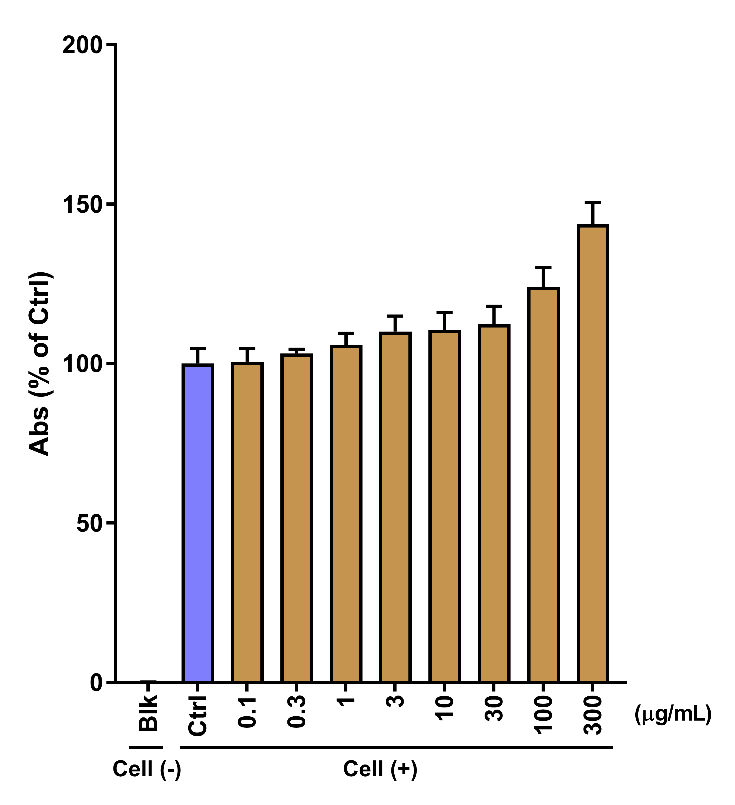


**Supplementary Figure 1.**

Optimization of experimental conditions for evaluating anti-inflammatory activity using HOK cells. **(A)** Assessment of cell density, IL-1β concentration, and assay duration (n = 3 wells). **(B)** Assessment of IL-1β concentration and assay duration (n = 3 wells). **(C)** Dose–response relationship between DCF and 1 ng/mL IL-1β–induced PGE2 production in HOK cells (n = 3 wells, IC_50_ = 0.31 nM). (D) Effect of representative HST (Lot# 392107100) on PGE2 production and cell viability in HOK cells (n = 3 wells). Black bars, medium; blue bars, medium; red bars, 1–30 ng/mL IL-1β; black symbols, medium + medium; blue symbols, medium + medium; red symbols, 1 ng/mL IL-1β + medium; yellow symbols, 1 ng/mL IL-1β + 0.01–30 nM DCF, brown bars: 0.1–300 μg/mL HST. Data are means ± SDs (error bars). Blk: blank; Ctrl: control.

**Figure S2.**


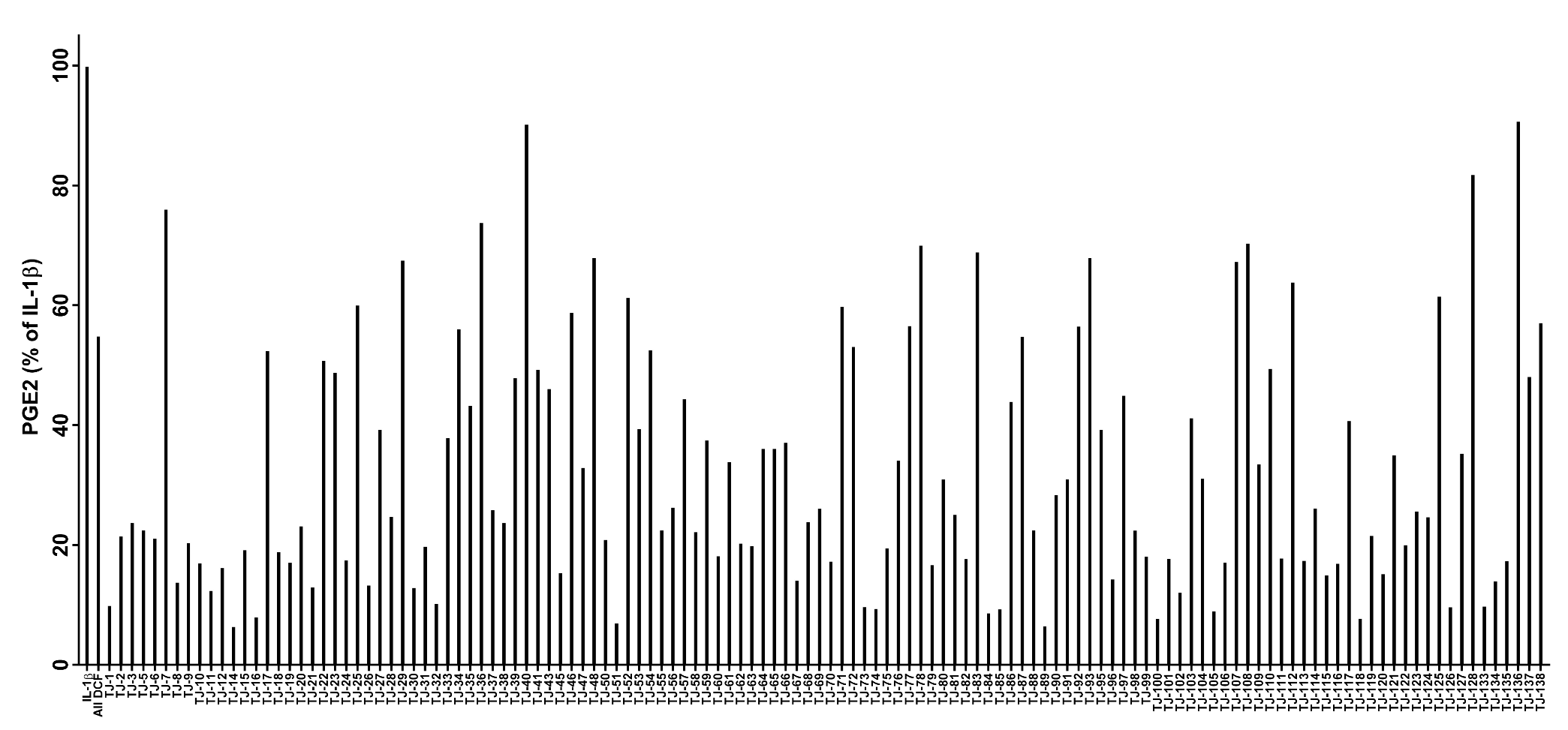


**Supplementary Figure 2.**

Evaluation of the detection capability of the cell-based bioassay for anti-inflammatory activity. Effects of 128 Kampo formulations on 1 ng/mL IL-1β–induced PGE2 production in HOK cells (IL-1β, n = 20 wells; all DCF, n = 20 wells; Kampo samples, n = 2 wells). “All DCF” represents the average measurement for DCF (0.3 nM) across all assay plates. All measurement values were standardized using the DCF control. DCF: diclofenac sodium.

**Table S3.** Inhibition ratios of PGE2 production (% of IL-1β) for 128 Kampo formulations (n = 2).

| **Sample** | **PGE2 (%)** |
| --- | --- |
| IL-1β | 100.00 |
| All DCF | 54.95 |
| TJ-1 | 9.99 |
| TJ-2 | 21.59 |
| TJ-3 | 23.79 |
| TJ-5 | 22.58 |
| TJ-6 | 21.17 |
| TJ-7 | 76.13 |
| TJ-8 | 13.87 |
| TJ-9 | 20.53 |
| TJ-10 | 17.12 |
| TJ-11 | 12.52 |
| TJ-12 | 16.30 |
| TJ-14 | 6.49 |
| TJ-15 | 19.32 |
| TJ-16 | 8.13 |
| TJ-17 | 52.54 |
| TJ-18 | 19.04 |
| TJ-19 | 17.19 |
| TJ-20 | 23.27 |
| TJ-21 | 13.14 |
| TJ-22 | 50.95 |
| TJ-23 | 48.90 |
| TJ-24 | 17.63 |
| TJ-25 | 60.06 |
| TJ-26 | 13.39 |
| TJ-27 | 39.36 |
| TJ-28 | 24.93 |
| TJ-29 | 67.59 |
| TJ-30 | 13.05 |
| TJ-31 | 19.92 |
| TJ-32 | 10.30 |
| TJ-33 | 37.98 |
| TJ-34 | 56.17 |
| TJ-35 | 43.41 |
| TJ-36 | 73.88 |
| TJ-37 | 25.96 |
| TJ-38 | 23.77 |
| TJ-39 | 48.05 |
| TJ-40 | 90.29 |
| TJ-41 | 49.38 |
| TJ-43 | 22.28 |
| TJ-45 | 37.56 |
| TJ-46 | 18.31 |
| TJ-47 | 33.95 |
| TJ-48 | 20.43 |
| TJ-50 | 20.03 |
| TJ-51 | 36.21 |
| TJ-52 | 36.21 |
| TJ-53 | 37.22 |
| TJ-54 | 14.17 |
| TJ-55 | 24.01 |
| TJ-56 | 26.25 |
| TJ-57 | 17.41 |
| TJ-58 | 46.23 |
| TJ-59 | 15.50 |
| TJ-60 | 58.93 |
| TJ-61 | 33.03 |
| TJ-62 | 67.99 |
| TJ-63 | 20.97 |
| TJ-64 | 7.08 |
| TJ-65 | 61.39 |
| TJ-66 | 39.48 |
| TJ-67 | 52.71 |
| TJ-68 | 22.62 |
| TJ-69 | 26.37 |
| TJ-70 | 44.53 |
| TJ-71 | 59.86 |
| TJ-72 | 53.24 |
| TJ-73 | 9.75 |
| TJ-74 | 9.50 |
| TJ-75 | 19.58 |
| TJ-76 | 34.17 |
| TJ-77 | 56.69 |
| TJ-78 | 70.11 |
| TJ-79 | 16.80 |
| TJ-80 | 31.15 |
| TJ-81 | 25.24 |
| TJ-82 | 17.75 |
| TJ-83 | 69.05 |
| TJ-84 | 8.77 |
| TJ-85 | 9.39 |
| TJ-86 | 44.07 |
| TJ-87 | 54.88 |
| TJ-88 | 22.64 |
| TJ-89 | 6.59 |
| TJ-90 | 28.47 |
| TJ-91 | 31.15 |
| TJ-92 | 56.59 |
| TJ-93 | 68.01 |
| TJ-95 | 39.41 |
| TJ-96 | 14.42 |
| TJ-97 | 45.08 |
| TJ98 | 22.55 |
| TJ-99 | 18.22 |
| TJ-100 | 7.82 |
| TJ-101 | 17.86 |
| TJ-102 | 12.21 |
| TJ-103 | 41.33 |
| TJ-104 | 31.23 |
| TJ-105 | 9.09 |
| TJ-106 | 17.22 |
| TJ-107 | 67.41 |
| TJ-108 | 70.46 |
| TJ-109 | 33.62 |
| TJ-110 | 49.56 |
| TJ-111 | 17.91 |
| TJ-112 | 63.96 |
| TJ-113 | 17.53 |
| TJ-114 | 26.27 |
| TJ-115 | 15.10 |
| TJ-116 | 17.05 |
| TJ-117 | 40.83 |
| TJ-118 | 7.86 |
| TJ-119 | 21.68 |
| TJ-120 | 15.29 |
| TJ-121 | 35.12 |
| TJ-122 | 20.15 |
| TJ-123 | 25.76 |
| TJ-124 | 24.79 |
| TJ-125 | 61.61 |
| TJ-126 | 9.78 |
| TJ-127 | 35.40 |
| TJ-128 | 81.89 |
| TJ-133 | 9.91 |
| TJ-134 | 14.12 |
| TJ-135 | 17.45 |
| TJ-136 | 90.78 |
| TJ-137 | 48.19 |
| TJ-138 | 57.18 |

DCF: diclofenac sodium.

**Table S4.** **Quantitative analysis of** **eight components**

|  | **peak area** | **content (µg/g)** |
| --- | --- | --- |
| [6]-Gingerol | 1148393 ± 293596 | 248 ± 72.48 |
| [8]-Gingerol | 273361 ± 43843 | 36 ± 5.92 |
| [10]-Gingerol | 268427 ± 22320 | 28.96 ± 2.38 |
| [6]-Shogaol | 748824 ± 179859 | 172.81 ± 45.98 |
| [8]-Shogaol | 129396 ± 24799 | 88.59 ± 8.35 |
| [10]-Shogaol | 170965 ± 26314 | 32.37 ± 5.07 |
| Neoglycyrol | 341874 ± 17894 | 21.33 ± 1.24 |
| Glycycoumarin | 1820304 ± 157953 | 88.59 ± 8.35 |

Data are means ± SDs of three lots (372200900, 382092900, 392165800)
